# Supplementary figures and images for: Atf3 links loss of epithelial polarity to defects in cell differentiation and cytoarchitecture
Source: PLoS Genet. 2018 Mar 1;14(3):e1007241. doi: 10.1371/journal.pgen.1007241 (PMC5849342; doi:10.1371/journal.pgen.1007241)

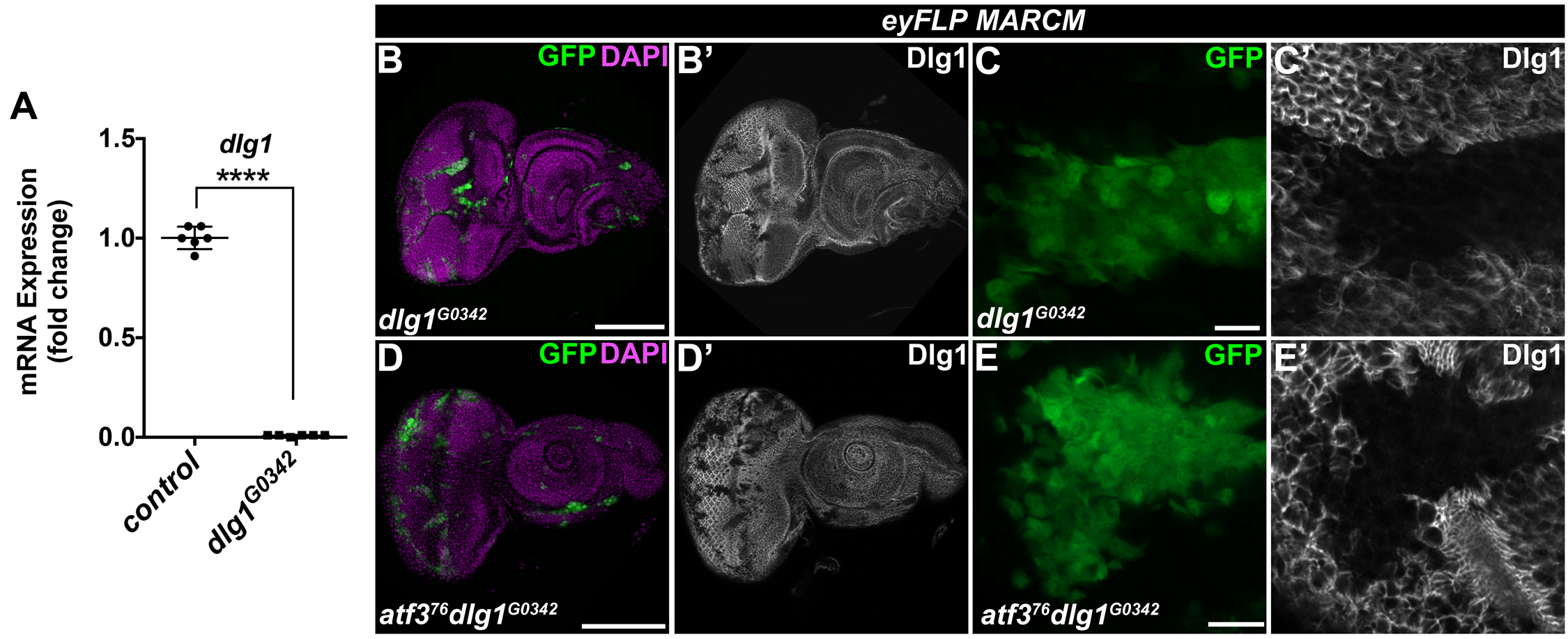

Supplement: S1 Fig — (A) No dlg1 transcript is detected in hemizygous dlg1G0342 larvae relative to control. (B-E) eyFLP-mediated mitotic recombination was used to generate clones (GFP) in the EADs of the indicated genotypes. Homozygous dlg1G0342 and atf376 dlg1G0342 EAD clones (B-C and D-E, respectively) are deficient for Dlg1 protein (B’-E’). qRT-PCR data are means of 6 biological replicates. Error bars depict 95% confidence interval; Unpaired Student’s t-tests assuming unequal variance were used to calculate p-values: **** = p<0.0001. Scale bars: 100 μm (B,D), 10 μm (C,E). (TIF) [file pgen.1007241.s001.tif]

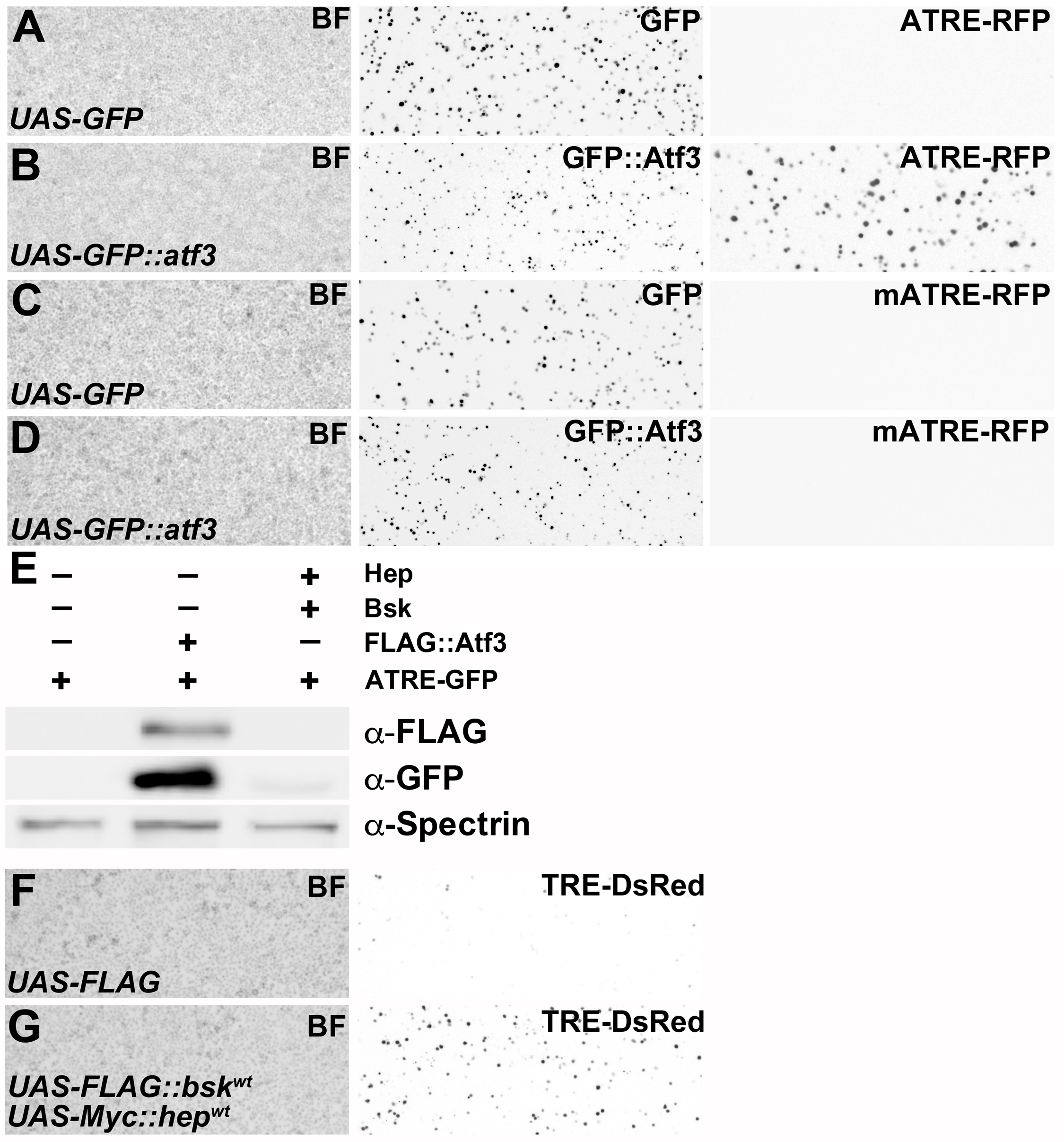

Supplement: S2 Fig — (A-B) In S2 cells, Atf3 induced the ATRE-RFP reporter compared to undetectable activity in control cells. (C-D) Atf3 did not activate a mutant mATRE-RFP reporter. (E-G) Expression of FLAG::Atf3 strongly induced an ATRE-GFP reporter in S2 cells. While co-expression of Hep/JNKK and Bsk/JNK induced an AP-1 reporter (TRE-DsRed) (F,G), it only weakly activated the ATRE reporter (E). α-Spectrin served as a loading control. (TIF) [file pgen.1007241.s002.tif]

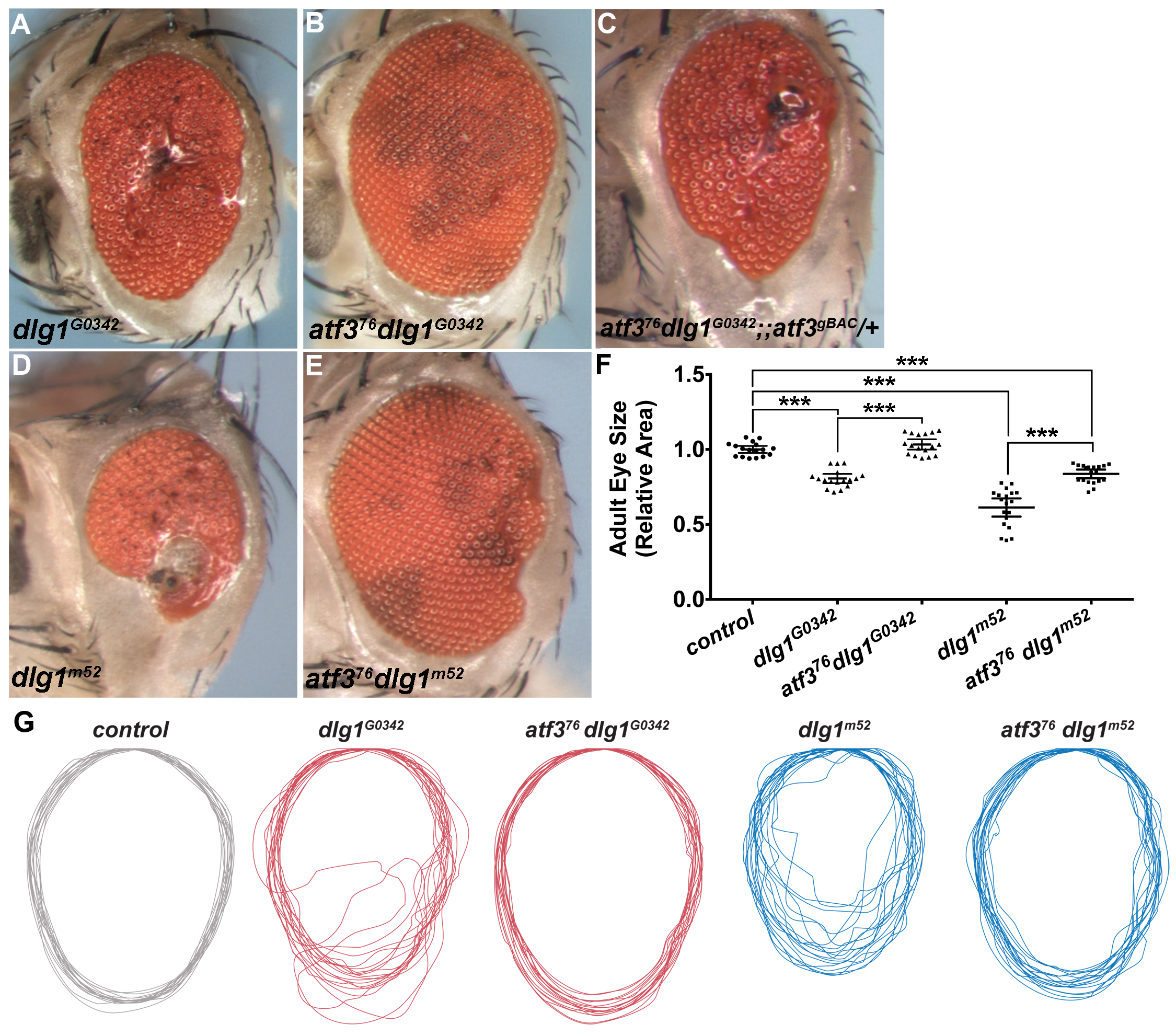

Supplement: S3 Fig — (A-G) eyFLP-mediated mitotic recombination was used to generate clones of the indicated genotypes in the eye. Homozygous dlg1G0342 or dlg1m52 EAD clones lead to smaller adult eyes of irregular shape (F,G) containing patches of undifferentiated tissue (A,D) relative to control (G). Adult eyes derived from atf376 dlg1G0342 or atf376 dlg1m52 mosaic EADs exhibit mild or no differentiation defects (B,E) as well as restored eye size and shape (F,G). A single copy of a genomic atf3gBAC reinstates differentiation defects to atf376 dlg1G0342 adult eyes (C). Outlines of adult eyes from the indicated genotypes are presented vertically aligned along their midline (G). Adult eye measurements were performed from at least 17 biological replicates. Error bars depict 95% confidence interval; Unpaired Student’s t-tests assuming unequal variance were used to calculate p-values: *** = p<0.001. (TIF) [file pgen.1007241.s003.tif]

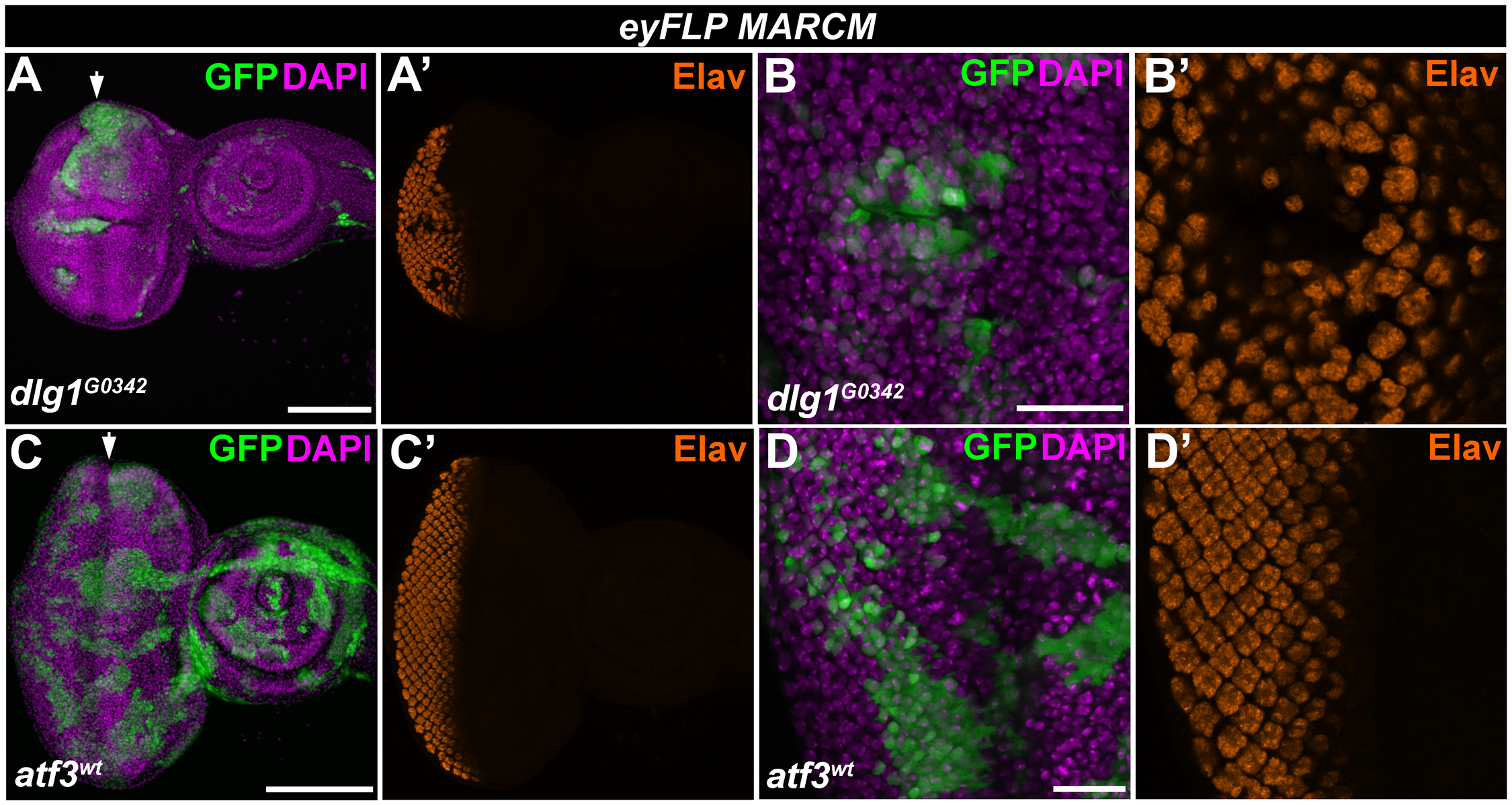

Supplement: S4 Fig — (A-D) eyFLP-mediated mitotic recombination was used to generate clones (GFP) in the EAD of the indicated genotypes. dlg1G0342 mutant cells located to the left of the morphogenetic furrow (arrow) are often Elav-negative (A,B) compared to a regular Elav staining pattern in the atf3wt clones (C,D) and the non-clonal neighbors. Discs were counterstained with DAPI. Micrographs are single confocal slices. All images show EADs 7 days after egg laying. Scale bars: 100 μm (A,C), 20 μm (B,D). (TIF) [file pgen.1007241.s004.tif]

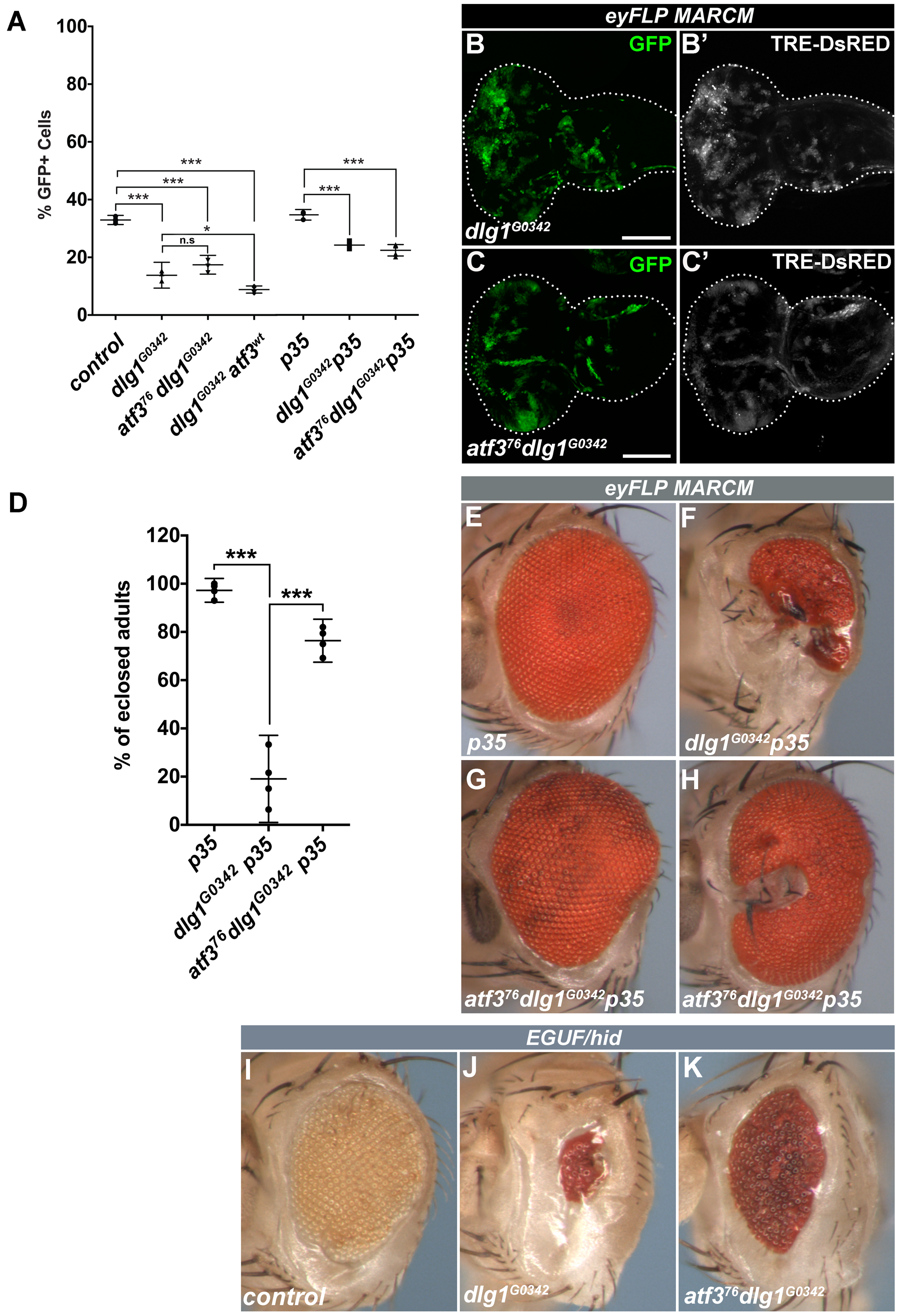

Supplement: S5 Fig — (A-H) eyFLP-mediated mitotic recombination was used to generate GFP-labeled clones of the indicated genotypes in the EAD. (A) The number of GFP+ cells in EADs bearing clones of the indicated genotypes was determined by flow cytometry. Relative to control (n = 4), dlg1G0342 (n = 3), atf376 dlg1G0342 (n = 4), and dlg1G0342 atf3wt (n = 5) clones were less abundant. dlg1G0342 atf3wt cells were less frequent relative to dlg1G0342 alone. Blocking apoptosis raised the relative abundance of dlg1G0342 (n = 7) and atf376 dlg1G0342 (n = 6) cells, but not to control (n = 4) levels. Error bars reflect the 95% confidence interval. Unpaired Student’s t-tests assuming unequal variance were used to calculate p-values: *p = 0.026, *** = p<0.001. (B-C) An AP-1 reporter (TRE-DsRed) serves as a readout of JNK pathway activity and is upregulated in dlg1G0342 (B’) and atf376dlg1G0342 (C’) EAD clones. (D) The eclosion rate of animals bearing atf376dlg1G0342p35 EAD was less than control but was four times higher than animals bearing dlg1G0342p35 EAD. Four biological replicates were used for each genotype. Unpaired Student’s t-tests assuming unequal variance were used to calculate p-values: *** = p<0.001. (E-H) Compared to control (E), adult eyes derived from dlg1G0342p35 EAD were very small, comprising mostly undifferentiated tissue (F), while those derived from atf376dlg1G0342p35 EAD exhibited only traces (G) or small patches (H) of defective photoreceptor differentiation. (I-K) The EGUF/hid technique was used to generate adult eyes comprised entirely of control (I), dlg1G0342 (J) and atf376dlg1G0342 (K) clonal tissue, as non-clonal cells were removed by expression of pro-apoptotic protein Hid. Compared to control (I), dlg1G0342 eyes were severely reduced in size with only traces of ommatidia left (J). atf376dlg1G0342 eyes were only mildly reduced relative to control and contained several rows of orderly arranged ommatidia (K). Micrographs (B,C) are projections of multiple confocal section [file pgen.1007241.s005.tif]

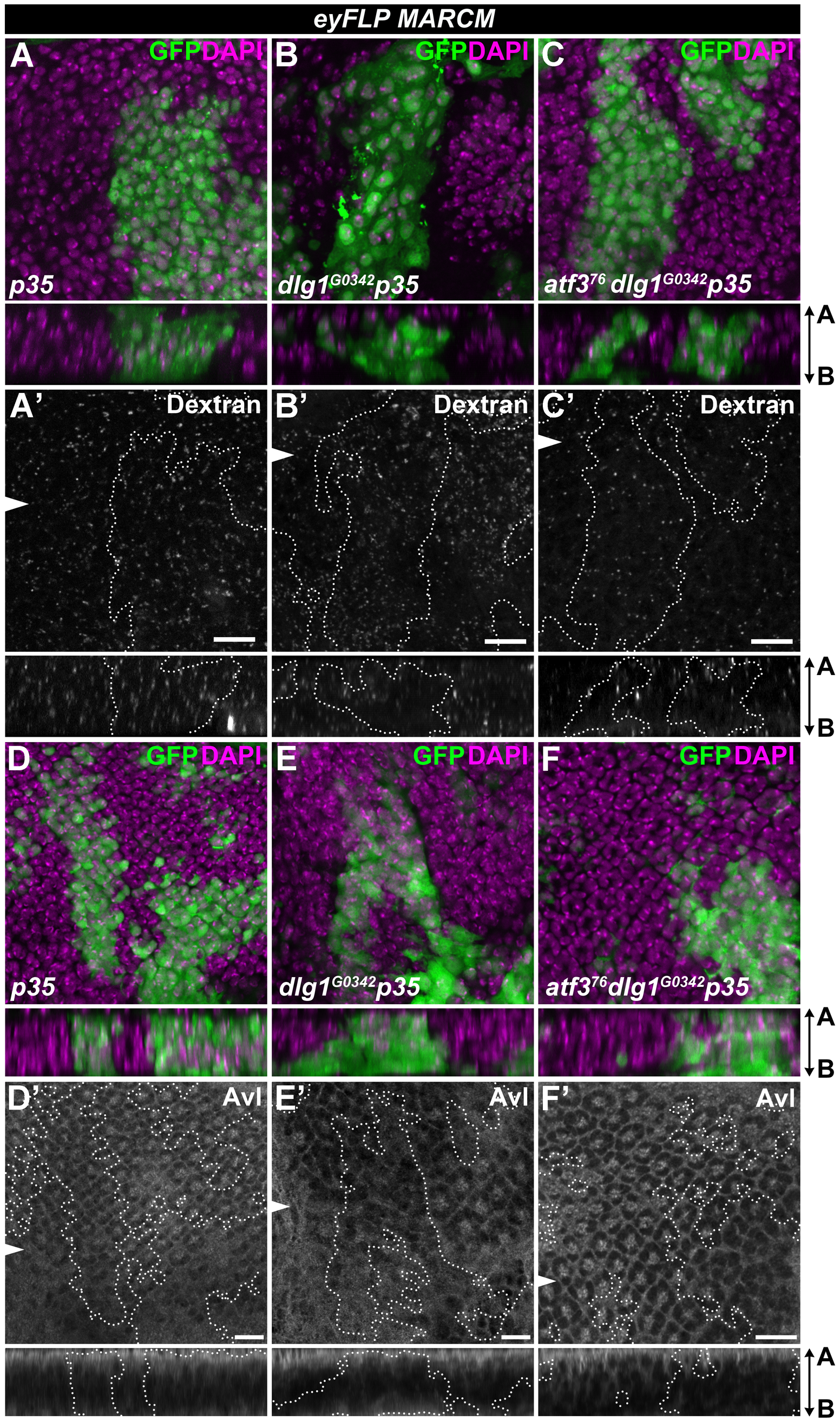

Supplement: S6 Fig — (A-F) eyFLP-mediated mitotic recombination was used to generate clones (GFP) in the EADs of the indicated genotypes. Apoptosis of mutant cells was reduced by p35 overexpression. (A-C) dlgG03421 and atf376dlg1G0342 clones show no disturbances in the uptake of fluorescently labeled dextran (B’- C’) compared to control clones (A’). (D-F) The regular pattern of the early endosomal marker Avl (D’) is disrupted in dlg1 mutant cells (E’), but restored in atf376dlg1G0342 double mutant clones (F’). Discs were counterstained with DAPI. White arrows indicate cross sections, which appear below the corresponding panels and are oriented apical side up. Clones are outlined by white dotted lines. All images show EAD 7 days after egg laying. Scale bars: 10 μm (A-F). (TIF) [file pgen.1007241.s006.tif]

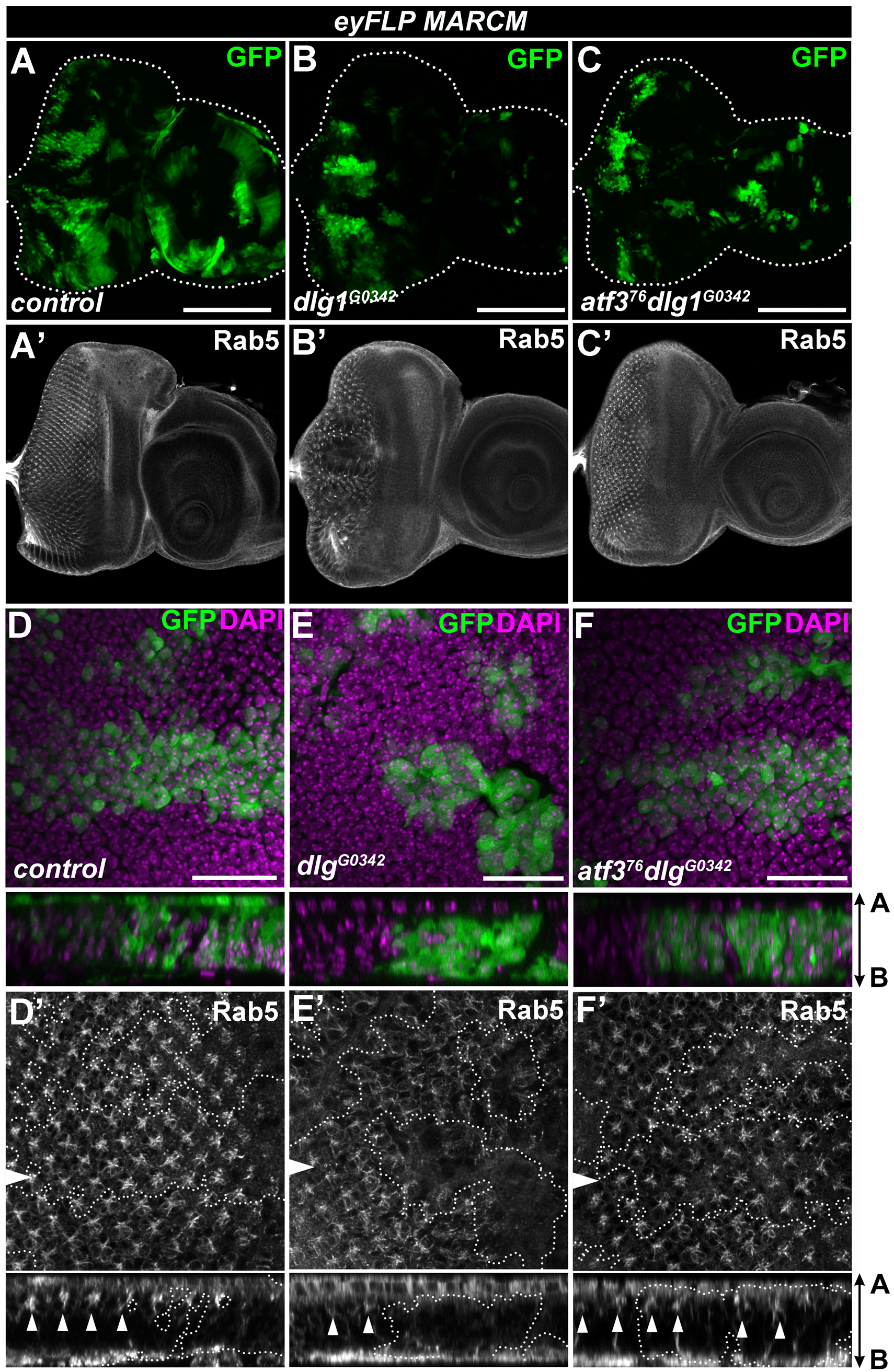

Supplement: S7 Fig — (A-F) eyFLP-mediated mitotic recombination was used to generate clones (GFP) in the EADs of the indicated genotypes. Lower levels and disturbed pattern of the early endosome marker Rab5 (B’,E’) observed in dlg1G0342 clones relative to surrounding and control tissue (A’,D’) were largely restored in atf376dlg1G0342 clones (C’,F’). Discs were counterstained with DAPI. In A-C, EADs and in D’-F’, clones are outlined with dotted white lines. White arrows indicate cross sections, which appear below the corresponding panels and are oriented apical side up. On the cross sections of D’-F’, arrowheads indicate the regular Rab5 accumulation in the photoreceptors. Micrographs are projections of multiple confocal slices (A-F). Scale bars: 100 μm (A-C), 20 μm (D-F). (TIF) [file pgen.1007241.s007.tif]

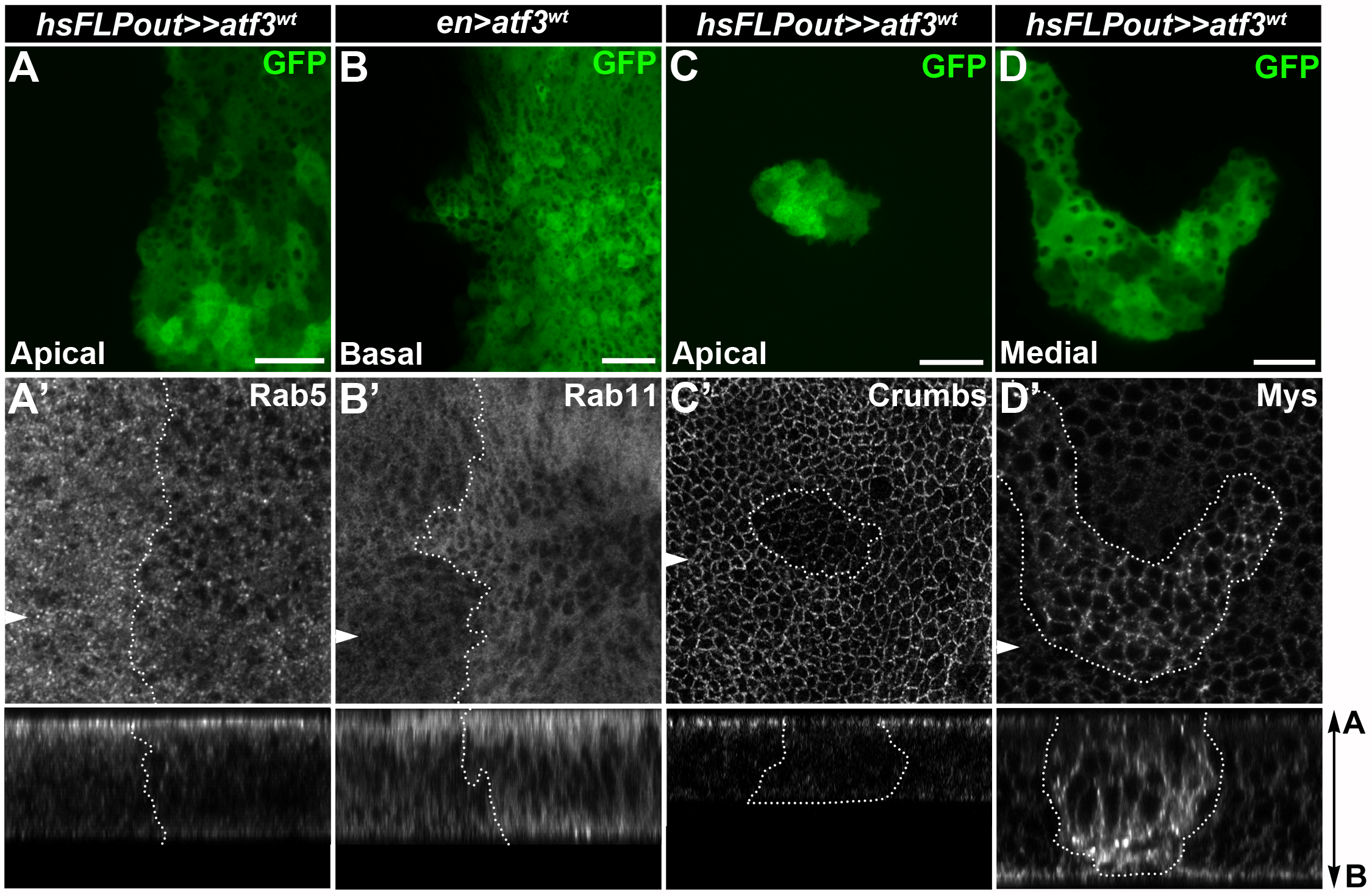

Supplement: S8 Fig — (A-B) Abundance of Rab5 vesicles (A’) was reduced in cells expressing Atf3 (hsFLPout>>atf3wt) (GFP), compared to surrounding tissue while the amount of Rab11 endosomes increased and showed a distinct basal accumulation in Atf3 overexpressing cells (en>atf3wt) (GFP) compared to wild type epithelium (B’). (C-D) Cross sections of immunostained wing imaginal disc clones expressing Atf3 (hsFLPout>>atf3wt) revealed lower levels of the polarity determinant Crumbs on the apical surface (C’). In contrast, the βPS integrin Myospheroid (Mys) appeared to be more abundant in Atf3 overexpressing cells and was distributed along the lateral membranes (D’). White dotted lines indicate clones (A,C,D) or the posterior compartment (B) in the wing disc. Arrowheads indicate cross sections, which appear below the corresponding panels and are oriented apical side up. Micrographs are otherwise single confocal slices. Scale bars: 10 μm (A-D). (TIF) [file pgen.1007241.s008.tif]

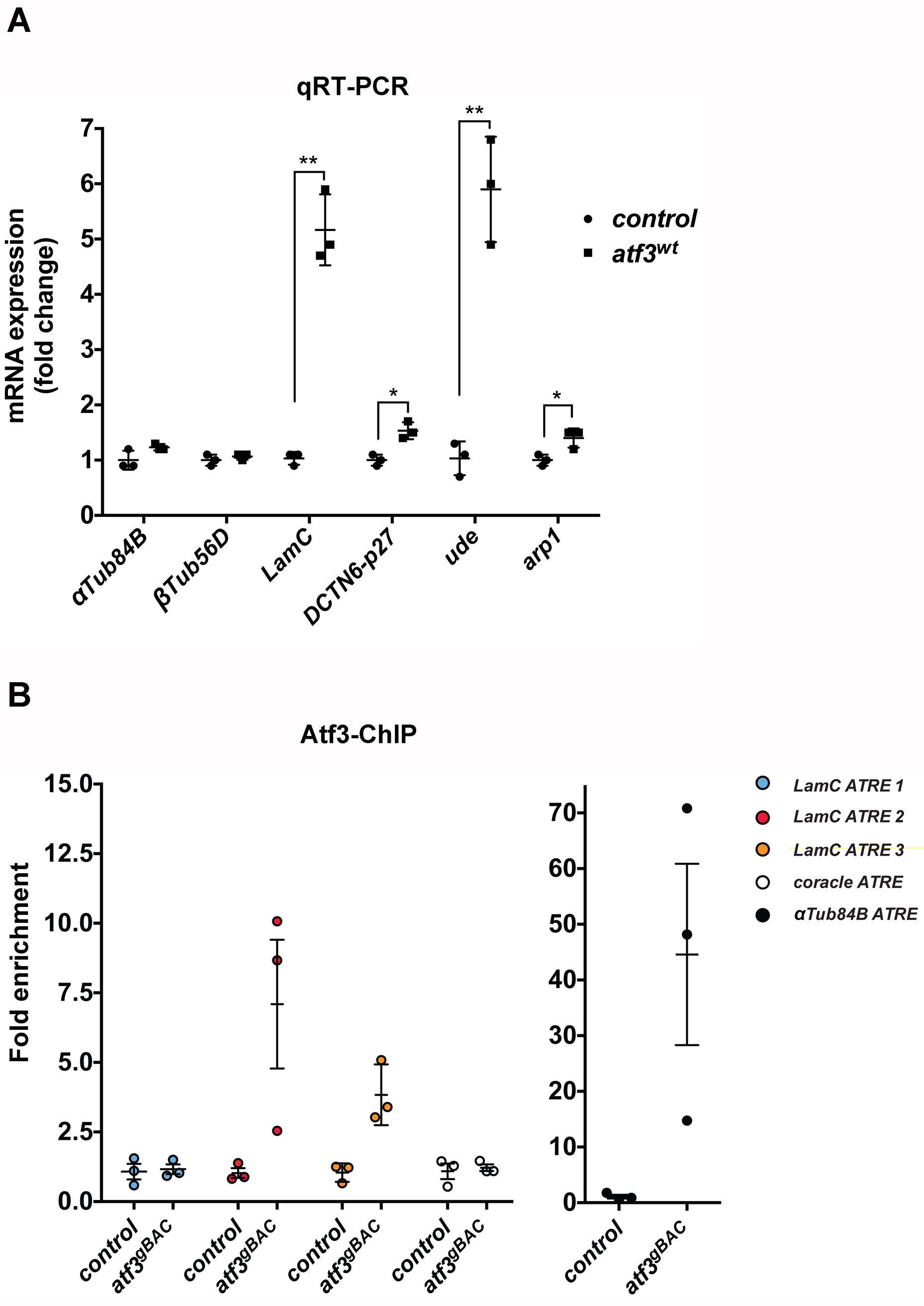

Supplement: S9 Fig — (A) Independent qRT-PCR from newly isolated mosaic EADs confirmed RNA-seq results showing enrichment of LamC, DCTN6-p27, ude and arp1 transcripts in EADs overexpressing Atf3 (n = 3) relative to control (n = 3) while αTub84B and βTub56D remained unchanged. (B) ChIP from third instar EADs followed by qPCR was used to quantify enrichment of the indicated Atf3 sites in atf3gBAC samples (n = 3) relative to w1118 control (n = 3). LamC and αTub84B were enriched in atf3gBAC samples compared to control. Note that LamC was bound by Atf3 at two of the three Atf3 sites within the gene. Atf3 did not bind the ATRE site in coracle which was occupied in samples from adults. Error bars indicate 95% confidence interval; Unpaired Student’s t-tests assuming unequal variance were used to calculate p-values: **p = 0.007 (LamC), *p = 0.018 (DCTN6-p27), **p = 0.008 (ude), *p = 0.024 (arp1). (TIF) [file pgen.1007241.s009.tif]

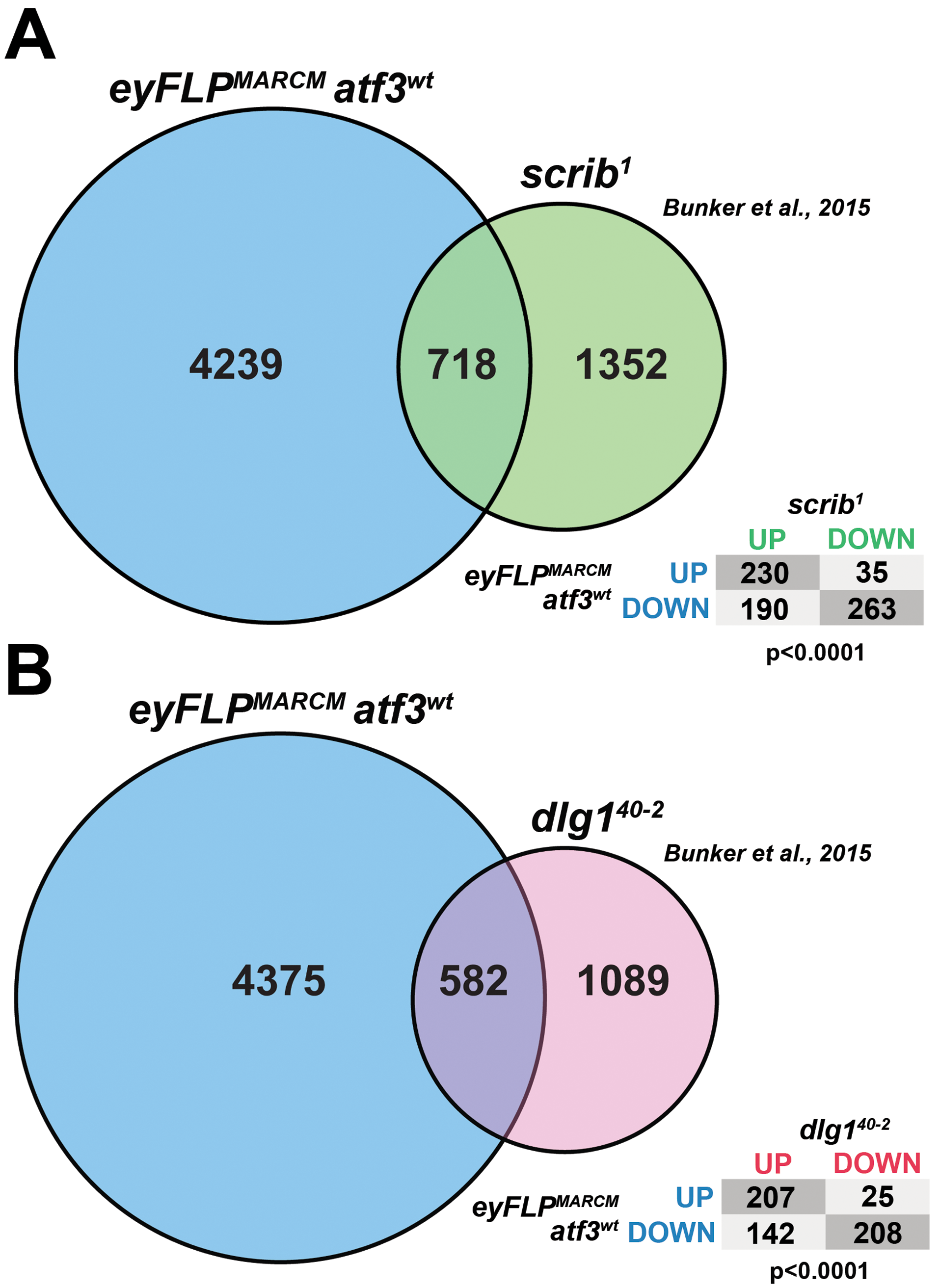

Supplement: S10 Fig — (A-B) Venn diagrams show the overlap between genes differentially regulated by a factor ≥1.5 in mosaic EADs overexpressing Atf3 (eyFLPMARCM atf3wt) and wing imaginal discs of scrib1 homozygous or dlg40-2/Y hemizygous mutant larvae. Contingency tables provide information about directionality of expression of shared transcripts between the two datasets and serve to calculate the significance of overlap using the one-tailed Fisher Exact Probability test. (TIF) [file pgen.1007241.s010.tif]

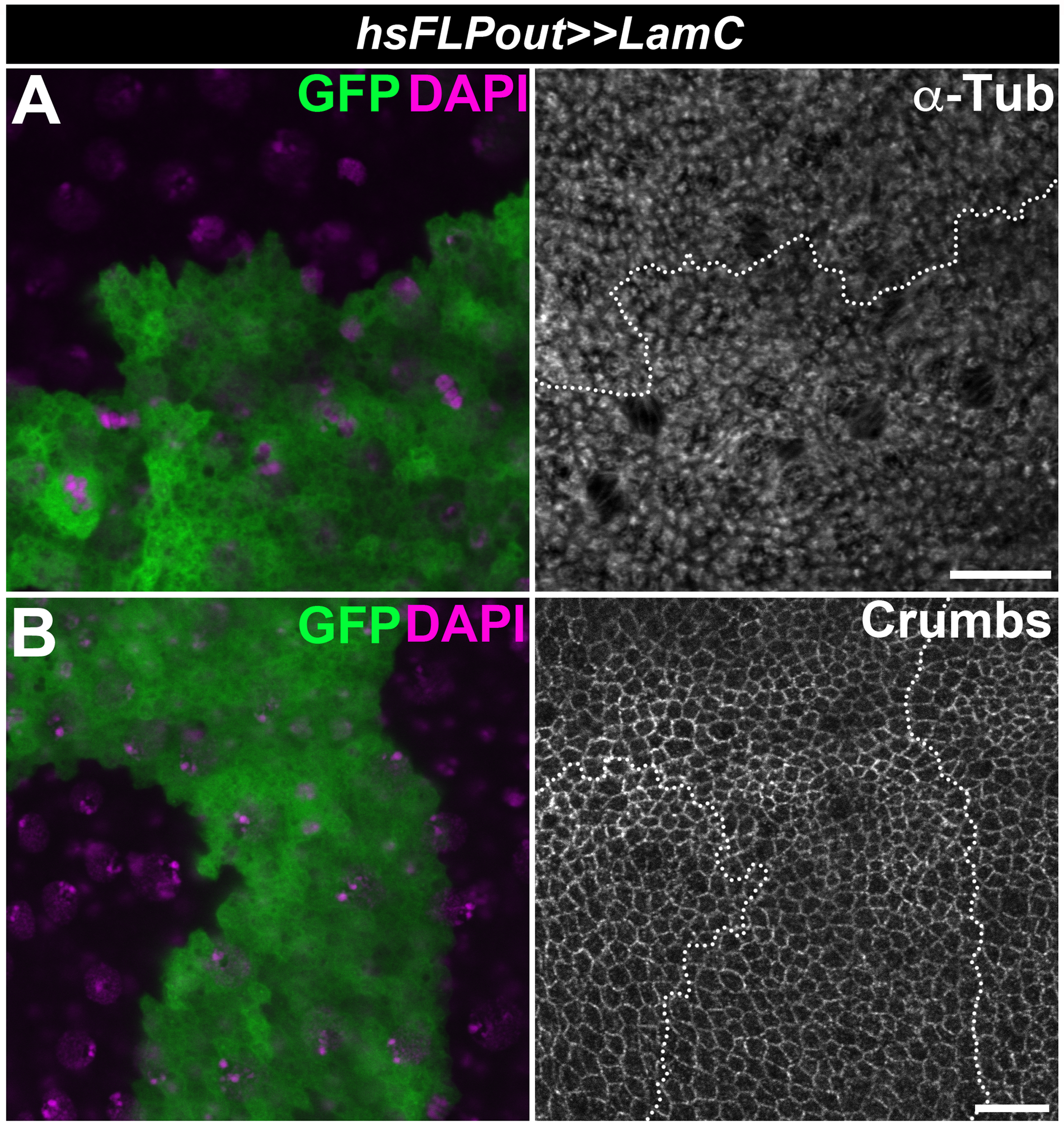

Supplement: S11 Fig — (A-B) Immunostaining for α-Tubulin (A) and Crumbs (B) is unchanged in wing imaginal disc clones (GFP) expressing wild type LamC (hsFLPout>>LamC). Discs were counterstained with DAPI. Clones are outlined with dotted white lines. White arrows indicate cross sections, which appear below the corresponding panels and are oriented apical side up. Scale bars: 10 μm (A,B). (TIF) [file pgen.1007241.s011.tif]
