# Supplementary material for: Atf3 links loss of epithelial polarity to defects in cell differentiation and cytoarchitecture
Source: PLoS Genet. 2018 Mar 1;14(3):e1007241. doi: 10.1371/journal.pgen.1007241 (PMC5849342; doi:10.1371/journal.pgen.1007241)
Supplement: S1 Table — (PDF) [file pgen.1007241.s012.pdf]

Table S1. Summary of oligonucleotides

Oligonucleotides used (5′ >3′ sequence)	
ATRE Reporter Oligos	
ATRE For_Mlu1	cgcgTTGAAAA**TGACGTCA**CTGGGCGGAGACAA**TCACGTCA**TCCAGCCGGCG<br>CGC**TTACGTCA**TTCATAATCGATGA**TGAGGTCA**TAACTAA
ATRE Rev_Not1	ggccTTAGTTA**TGACCTCA**TCATCGATTATGAA**TGACGTAA**GCGCGCCGGCT<br>GGA**TGACGTGA**TTGTCTCCGCCCAG**TGACGTCA**TTTTCAA
mATRE_ For_Mlu1	cgcgAGAGTCGACATGAGAGTCGACATGAGAGTCGACATGAGAGTCGACATG
mATRE_Rev_Not1	ggccCATGTCGACTCTCATGTCGACTCTCATGTCGACTCTCATGTCGACTCT
qRT-PCR	
rp49 For	tcctaccagcttcaagatgac
rp49 Rev	cacgttgtgcaccaggaact
atf3 iQ For	tggtggacatgctgaaatcgca
atf3 iQ Rev	atgctgctggtcaatcacgttg
dlg1 iQ For	gctgctcagcgtgaacaatgtcaa
dlg1 iQ Rev	gggcagcctgttgtttcaactctt
LamC ATRE 1 ChIP For	agctttcgcaaacaaatgtgagtcg
LamC ATRE 1 ChIP Rev	cctatttgcctgagtaatcccttatcg
LamC ATRE 2 ChIP For	gagtgacagagacagacagagaacg
LamC ATRE 2 ChIP Rev	cagcgaaagagtcgagagagtttcc
LamC ATRE 3 ChIP For	ggaggtcagtgctatatacactcgattta
LamC ATRE 3 ChIP Rev	ctgtctcaacacacgctgtgagaa
coracle ATRE ChIP For	gctcttgatattatgccttagctggtgc
coracle ATRE ChIP Rev	cgaattgtcgatagcgcatagcgatt
αTub84B ATRE ChIP For	gctgaaaagaaatttgtgtgggcaaa
αTub84B ATRE ChIP Rev	taagagccattcctcattgcgaaca
ecd iQ For	acccaagaagctctacaagccgaa
ecd iQ Rev	acctttgctgtcagtcctgtggaa
αTub84B iQ For	tcatagccggcagttcgaacgtat
αTub84B iQ Rev	acaccagcctgaccaacatggata
βTub56D iQ For	ccagaacaagaacagctcctac
βTub56D iQ Rev	cagtggagttgccgatgaa
arp1 iQ For	gctggagggcgacatattcgttg
arp1 iQ Rev	ggtgacaatgccgtgctccatg

dynactin iQ For	atataaattccttctcaccttcgtg
dynactin iQ Rev	cggtggcatattcctctatga
ude iQ For	gacatggagaaggcgttagag
ude iQ Rev	gctctctgtcttggcattct
LamC iQ For	caacctggagaacgagaaca
LamC iQ Rev	ctcatagaccgccttcagattag
Cloning primers	
atf3 cDNA For	caccatgttcaattccaacataccggcctcc
atf3 cDNA Rev	tcacgccaggcaactgcc
