# Supplementary material for: Atf3 links loss of epithelial polarity to defects in cell differentiation and cytoarchitecture
Source: PLoS Genet. 2018 Mar 1;14(3):e1007241. doi: 10.1371/journal.pgen.1007241 (PMC5849342; doi:10.1371/journal.pgen.1007241)
Supplement: S2 Table — (PDF) [file pgen.1007241.s013.pdf]

Table S2. Summary of fly crosses

**ChIP-seq**		
♀ *y w*	x	♂ *w^{1118}*
♀ *y $atf3^{76}$ w/ FM7i, P{ActGFP}JMR3;; $atf3_{g}^{BAC}$/TM6B*	x	♂ *w^{1118}*
**ChIP**		
♀ *w^{1118}*	x	♂ *y w*
♀ *w^{1118}*	x	♂ *y $atf3^{76}$ w;; $atf3_{g}^{BAC}$/TM6B*
**Wing Disc Expression**		
♀ w; *en-GAL4; $atf3_{g}^{BAC}$/TM6B*	x	♂ *w^{1118}*
♀ w; *en-GAL4; $atf3_{g}^{BAC}$/TM6B*	x	♂ *w; UAS-dlg^{RNAi}*
♀ w; *en-GAL4, UAS-mCherry ; $atf3_{g}^{BAC}$/TM6B*	x	♂ *w; UAS-$scrib^{RNAi}$*
♀ w; *en-GAL4; $atf3_{g}^{BAC}$/TM6B*	x	♂ *w; UAS-hep^{wt}, TRE-dsRED*
♀ w; *en-GAL4; $atf3_{g}^{BAC}$/TM6B*	x	♂ *w;; UAS-$aPKC^{CAAX}$*
♀ w; *en-GAL4; $atf3_{g}^{BAC}$/TM6B*	x	♂ *w;; UAS-$aPKC^{CAAX}$, UAS-bsk^{DN}*
♀ w; *ATRE-GFP, UAS-mCherry; dpp-GAL4/TM6B*	x	♂ *w; UAS-$atf3^{wt}$*
♀ w; *ATRE-GFP, UAS-mCherry; dpp-GAL4/TM6B*	x	♂ *w; UAS-hep^{wt}*
♀ w; *ATRE-GFP, UAS-mCherry; dpp-GAL4/TM6B*	x	♂ *w;; UAS-$aPKC^{CAAX}$*
♀ w; *ATRE-GFP, UAS-mCherry; dpp-GAL4/TM6B*	x	♂ *w;; UAS-yki^{act}*
**Mosaic analysis**		
♀ *hsFLP; act>y^{+}>GAL4, UAS-GFP (hsFLPout>>)*		
♂ *w^{1118}*		
♂ *w; UAS-$atf3^{wt}$*		
♂ *w; UAS-LamC*		
♂ *eyFLP FRT19A, tubGAL80; act>y^{+}>GAL4, UAS-GFP (eyFLP MARCM)*		
♀ *y w FRT19A*		
♀ *y w $dlg1^{G0342}$ FRT19A / FM7i, P{ActGFP}JMR3*		
♀ *y $atf3^{76}$ w FRT19A / FM7i, P{ActGFP}JMR3*		
♀ *y $atf3^{76}$ w $dlg1^{G0342}$ FRT19A / FM7i, P{ActGFP}JMR3*		
♀ *y w $dlg1^{m52}$ FRT19A / FM7i, P{ActGFP}JMR3*		
♀ *y $atf3^{76}$ w $dlg1^{m52}$ FRT19A / FM7i, P{ActGFP}JMR3*		
♀ *y w $dlg1^{G0342}$ FRT19A / FM7i, P{ActGFP}JMR3; UAS-$atf3^{wt}$*		
♀ *y w FRT19A; UAS-p35*		
♀ *y w $dlg1^{G0342}$ FRT19A / FM7i, P{ActGFP}JMR3; UAS-p35*		

♀ *y atf3*^{76} *w dlg1*^{G0342} *FRT19A / FM7i, P{ActGFP}JMR3; UAS-p35*
♂ *eyFLP FRT19A, tubGAL80; act>y*$^{+}$*>GAL4, UAS-myrRFP (eyFLP MARCM)*
♀ *y w dlg1*^{G0342} *FRT19A / FM7i, P{ActGFP}JMR3;; atf3*^{gBAC}*/TM6B*
♀ *y atf3*^{76} *w dlg1*^{G0342} *FRT19A / FM7i, P{ActGFP}JMR3;; atf3*^{gBAC}*/TM6B*
♀ *y w dlg1*^{G0342} *FRT19A / FM7i, P{ActGFP}JMR3;; UAS-bsk*^{DN}*, atf3*^{gBAC}*/TM6B*
♂ *GMR-hid y w* FRT19A; ey-GAL4, UAS-FLP (EGUF/hid)*
♀ *y w FRT19A*
♀ *y w dlg1*^{G0342} *FRT19A / FM7i, P{ActGFP}JMR3*
♀ *y atf3*^{76} *w dlg1*^{G0342} *FRT19A / FM7i, P{ActGFP}JMR3*
♀ *eyFLP; act>y*$^{+}$*>GAL4, UAS-GFP; FRT82B, tubGAL80 (eyFLP MARCM)*
♂ *w;; FRT82B*
♂ *w; UAS-atf3*^{wt}*; FRT82B*
